# Supervisor Bottom-Line Mentality, Performance Pressure, and Workplace Cheating: Moderating Role of Negative Reciprocity

**DOI:** 10.3389/fpsyg.2022.801283

**Published:** 2022-05-30

**Authors:** Komal Kamran, Akbar Azam, Mian Muhammad Atif

**Affiliations:** FAST School of Management, National University of Computer and Emerging Sciences, Islamabad, Pakistan

**Keywords:** supervisor bottom-line mentality, negative reciprocity belief, performance pressure, displaced aggression, workplace cheating

## Abstract

Employee cheating at the workplace has reached epidemic proportions and is putting a significant dent on the revenues of corporations. This study evaluates workplace cheating behavior as a consequence of supervisor bottom-line mentality with performance pressure as the mediating mechanism. Most importantly, it scrutinizes the moderating function of negative reciprocity belief in the relation between bottom-line mentality, performance pressure, and cheating in a moderated-mediation model, through the lens of displaced aggression theory. We systematically conduct time-lagged studies in two different populations (Pakistan and United States). Data analysis reveals that (1) bottom-line mentality positively influences workplace cheating behavior through performance pressure and (2) negative reciprocity moderated this indirect relationship. Theoretical and practical implications are discussed.

## Introduction

Business managers are constantly focusing on profitability, in order to deliver on “shareholder value.” The phenomenon has been termed as *bottom-line mentality* (BLM), which is a “one-dimensional thinking that revolves around securing bottom-line outcomes to the neglect of competing priorities” (Greenbaum et al., 2012, p. 344). Research evidence shows that such an emphasis on bottom-line attainment leads to enhanced organizational performance and employee productivity (Friedman, 2007), elevated employee task performance (Babalola et al., 2020b), increased shareholder value (Cushen, 2013), and organizational success (Davidsson et al., 2009). However, these individuals function with a tunnel vision by focusing primarily on financial outcomes—neglecting all other aspects, such as ethical adherence, employee morale, experience, and commitment (Wolfe, 1988; Estes and Estes, 1996; Babalola et al., 2020b).

Managers possessing BLM emphasize solely on their own success and survival in the organization’s competitive climate, such that they pay little or no attention to the welfare of other stakeholders (Bonner et al., 2017). Recent high-profile corporate scandals (e.g., Volkswagen and Wells Fargo) uncover the dark side of this one-dimensional thinking by providing anecdotal evidence for dysfunctional outcomes resulting from BLM (Babalola et al., 2020a). In fact, Quade et al. (2020) reveal that managers possessing high BLM may have a negative influence on an organization’s bottom-line attainment due to poor quality of leader–member exchange with their subordinates. Additionally, research indicates that SBLM may have adverse effects on employee’s organizational commitment and work–family conflict (Quade et al., 2021).

More recent studies have further explored the dysfunctional employee outcomes of BLM. Zhang et al. (2022) provide evidence for a curvilinear relationship between leader BLM and employee work performance such that its lower intensity may increase employee work performance, while its moderate-to-high intensity tends to have detrimental effects on employee work performance. It has also been linked with reduced levels of employee innovation as a result of psychological contract breach (Liu et al., 2022). Moreover, it leads to unethical pro-organizational behavior as a result of job insecurity among employees (Zhang et al., 2021). Even in work teams, supervisor’s exclusive focus on bottom-line outcomes triggers avoidance goal orientation, which negatively influences team performance (Lin et al., 2022). Furthermore, Babalola et al. (2022b) suggest that due to an intensely competitive work environment created by SBLM, employees may thrive at work but are highly likely to experience insomnia at home.

Moreover, findings from extant literature indicate that BLM has a significant positive association with employee cheating behavior (Farasat et al., 2020), abusive supervision (Mawritz et al., 2017), subordinates’ knowledge hiding (Li and Cheng, 2022), unethical pro-organizational behavior (UPB; Babalola et al., 2020b; Farasat and Azam, 2020; Zhang et al., 2021), and unethical pro-leader behavior (ULB; Mesdaghinia et al., 2019). Hence, it is evident that BLM may temporarily enhance the financial health of the organization but that comes with a huge long-term cost, mainly in terms of undesirable and unethical employee conduct.

Due to the dual nature of BLM outcomes, researchers are devoting their efforts to investigate the mechanisms through which positive consequences are maximized while negative ones are reduced. The current research extends the empirical literature by focusing on how and when SBLM influences an important unethical behavior, i.e., employee cheating. We study the mediating role of performance pressure on the association between SBLM and subordinates’ cheating behavior. Workplace cheating includes acts such as falsifying revenue figures; inflated invoicing; misrepresenting work-hours; theft of customer identity; deceitful expense claims; and payroll fraud (Gill et al., 2013). Incidents of such “infidelity” at the workplace are pervasive and have drastically grown in number. In fact, a staggering 33% of surveyed firms have reported cheating instances (PwC, 2014). Due to occupational fraud, a typical organization witnesses a drop of 5% in its revenue, which means a loss of $3.5 trillion globally (Ratley, 2014). According to Coffin (2003), employee theft and fraud account for approximately 20% of all business failures. These alarming statistics call for extensive research on why employees cheat and how this unethical behavior may be managed.

To that end, we propose that workplace cheating is an outcome of undue performance pressure among employees, stimulated by managers who are driven by bottom-line pursuits. By creating a highly competitive work environment and tying remuneration and rewards to bottom-line attainment, these managers unknowingly promote employee performance pressure (Prouska et al., 2016b). In such a situation, employees anticipate a threat to their self-interest and may resort to inflicting harm to the organization by cheating for self-preservation (Mitchell et al., 2018). Employees generally perceive managers as agents of the organization and may hold the organization responsible for the BLM-related actions of their supervisors. Therefore, they justify cheating in response to the performance pressure they undergo due to SBLM. Previous researchers have examined the impact of performance pressure on several employee outcomes such as enhanced work performance (Eisenberger and Aselage, 2009), increased innovativeness (Sitkin et al., 2011), unethical decision making (Mumford et al., 2006), inventory loss (Jensen et al., 2019), and workplace cheating (Mitchell et al., 2018). However, there is a dearth of investigations exploring its antecedents. This is the first study to identify SBLM as a precursor of performance pressure and link it to subordinates’ cheating behavior. Thus, one of the main contributions of this research is to study the mediating function of performance pressure in the association between SBLM and cheating.

Prior research has shown that SBLM may lead to employees making unethical choices. However, due to individual differences, not all subordinates working under high BLM leaders exhibit the same level of unethical behavior (Farasat et al., 2020). Some of the personal characteristics investigated in previous studies include: employee conscientiousness and core self-evaluations (Greenbaum et al., 2012); moral identity (Mesdaghinia et al., 2019); moral disengagement and power-distance orientation (Zhang et al., 2020); and employee entitlement (Farasat et al., 2020). However, the integral role of negative reciprocity has not been recognized in extant literature. This is the first study to theorize that negative reciprocity orientation of employees may influence the intensity of association between SBLM and unethical behavior like cheating. When employees high in negative reciprocity orientation experience performance pressure as a result of SBLM, they attempt to seek vengeance for this treatment. According to the theory of displaced aggression (Dollard et al., 1939), they are likely to take revenge from more available targets (in this case, the organization), instead of the initiator of mistreatment due to the fear of counter-retaliation. Hence, we suggest that employees possessing stronger negative reciprocity endorsement are more likely to indulge in unethical behavior as a response to performance pressure induced by high BLM.

**Figure 1:**
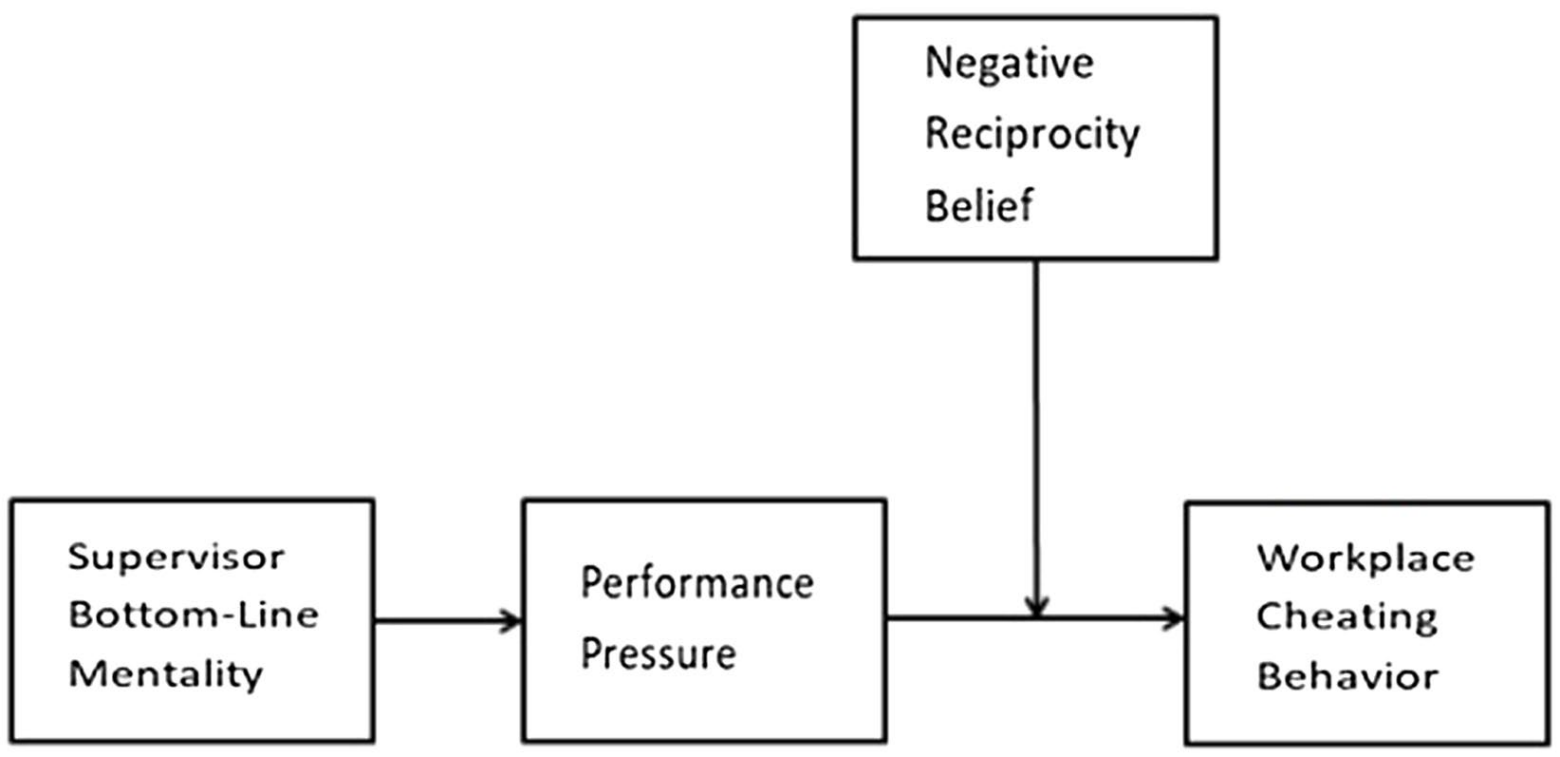
Conceptual model.

To recapitulate, this study aims to test whether SBLM is positively associated with employee performance pressure and whether performance pressure mediates the relationship between BLM and employee cheating behavior. It also builds on the “person–situation interactionist” perspective by proposing a moderated mediation model in which the indirect effect of SBLM on cheating, through performance pressure, is moderated by negative reciprocity orientation of employees (Figure 1).

## Theoretical Rationale and Hypotheses

### Supervisor BLM and Performance Pressure

Supervisors high in BLM emphasize on the significance of bottom-line achievement over any other competing value (Callahan, 2007). They expect their subordinates to exert all their efforts to contribute toward bottom-line success (Mawritz et al., 2017). Their behavior explicitly shows that bottom-line success is of paramount importance as they tend to reward employees who attain the bottom line and penalize those who fail to do so (Greenbaum et al., 2012). These employees get mentally preoccupied with work so as to direct their attention toward achieving stellar performance (Babalola et al., 2020a). In such “*do or die”* situations, an immense amount of performance pressure is likely to build up on the shoulder of employees, especially in today’s competitive and resource-constrained business environment.

Performance pressure is “a discomforting perception of the necessity for high performance” (Eisenberger and Aselage, 2009, p. 96), which arises due to any factor(s) that emphasizes on the significance of showing high performance on any specific occasion (Baumeister, 1984). It is the perception that attaining high levels of performance and achieving targets is highly important (Zhang et al., 2017). This can be attributed to the rewards, punishments, and resulting competition associated with these performance expectations (Prouska et al., 2016a). Similarly, according to Mitchell et al. (2018), performance pressure develops when employees are given the signal that failing to achieve performance-related goals may result in grave consequences. Performance pressure is explained as an external force exerted on employees to enhance their work performance and produce superior results for the organization (Gardner, 2012b).

Further, workers’ relationship with their organization is perceived as social exchange (Blau, 1964), wherein they are obligated to strive hard toward organizational goals in exchange for support, rewards, and compensation (Cropanzano and Mitchell, 2005). This boosts employee motivation to work hard and be creative so as to produce beneficial results for the organization (Rousseau, 1997). As a result of excessive performance demands on part of the organization, employees feel increasingly pressurized to elevate their performance into preserving their exchange relationship (Mitchell et al., 2018). The strength of the employees’ relationship with their organization, thus, would rely on employees enhancing their performance (Bernerth et al., 2016). In a similar fashion, SBLM places excessive behavioral expectations on employees to deliver superior financial outcomes for the organization (Babalola et al., 2020a). These compelling demands to act in a specific way lead to increased performance pressure among the subordinates (Baucus, 1994), to maintain the employee–organization relationship.

We propose that BLM managers, by associating performance with critical consequences, urge employees to achieve stretch targets to ensure bottom-line success and therefore foster performance pressure among them. Such leaders constantly communicate the cardinal importance of attaining bottom-line objectives to their subordinates (Quade et al., 2020). As a trickle-down effect, employees tend to mimic their supervisor’s BLM and are prompted to function with the same one-dimensional approach (Greenbaum et al., 2012). For those who do not contribute sufficiently toward the desired results are considered to be non-conformists and a hindrance—who should be prepared for deprecatory treatment (Mawritz et al., 2017) or other negative disciplinary actions such as being terminated, or deprived of career progression or accolades (Mesdaghinia et al., 2019). As the inability to secure bottom-line objectives may weaken the employee’s social standing in the organization (Mitchell et al., 2018), we posit that they are likely to experience a discomforting perception about the obligation to meet their manager’s bottom-line expectations. Thus, it is evident that the conditions created by SBLM become a perfect breeding ground for performance pressure to develop among their subordinates. This leads to the following hypothesis:

*Hypothesis 1*: SBLM has a positive relationship with subordinate’s performance pressure.

### Performance Pressure and Workplace Cheating Behavior

Managers exert performance pressure on workers to motivate them to perform well (Eisenberger and Aselage, 2009) and exhibit more creativity (Sitkin et al., 2011). However, research has demonstrated that performance pressure also increases unethical decision making (Mumford et al., 2006), workplace stress (Gardner, 2012a), unethical pro-organizational behavior (Chen and Chen, 2021), and cheating (Mitchell et al., 2018). In particular, we focus on the effect of performance pressure on workplace cheating behavior.

Workplace cheating is an unethical behavior defined as “accruing benefits to the self that violates accepted standards or rules” (Shu et al., 2011). According to Jones (1991), cheating refers to acts that may be illegal and/or against societal moral values. The emphasis of such behavior is on self-interest—the actor creates an unfair advantage specifically for the self. Moreover, cheating results in financial rewards or other favorable outcomes that the actor is not entitled to gain from the organization (Mitchell et al., 2018). Hence, employee cheating behavior is a deliberate moral transgression aimed at serving one’s personal interests.

According to Rigdon and D’Esterre (2015), employees adopt different approaches to cheating for self-gain: They may falsify their own work performance and they may misrepresent the work performance of their coworkers. Results from their experimental research suggest that individuals under competitive pressure are more inclined toward lying about themselves rather than harming others. As BLM tends to induce a viciously competitive work environment for employees putting them under performance pressure, we choose the characterization of workplace cheating where employees misrepresent work-related information to boost their own achievement levels. This characterization is validated by Mitchell et al. (2018) in their process of a developing a measure for cheating behavior. Based on data gathered for critical cheating-related incidents, three main themes emerged in their study: (a) acts where the individual creates an unfair advantage for the self, (b) self-interested behavior aimed at sabotaging others, and (c) actions intended to harm the organization but without self-gain. While the second theme relates to coworker social undermining and the third one points toward workplace deviance, only the first one (representing 50 % of the responses) was considered as cheating for personal benefit.

Based on the conceptualization of performance pressure, it influences individual perceptions about negative outcomes associated with the inability to achieve high-performance targets (Eisenberger and Aselage, 2009), and such a situation may result in cheating for self-interest (Van Yperen et al., 2011). Furthermore, performance pressure has been found to increase a feeling of helplessness and constriction (Sharma and Kirkman, 2015) along with fatigue and anxiety (Ellis, 2006). The resulting powerlessness and stress trigger a self-protection mode among the employee, as a result of which there is an increased predisposition to cheat (Mitchell et al., 2018). Schwartz (1987) posits that when a person’s well-being is threatened, self-interest motives are activated, leading toward self-preservation, which is an essential component of human behavior. Indeed, performance pressure is one of the most salient stressors that generates a visceral reaction toward self-serving acts (Wang and Murnighan, 2011).

Employees perceive performance pressure as a threatening experience, as it may underscore insufficiencies of existing efforts, be critical in maintaining their position in the organization, and have a role in their acceptance among social groups (Mitchell et al., 2018). This scenario is conducive to a self-preserving state of anger developing among the employees (Leary et al., 2006). Anger helps individuals to deal with the threat, allowing them to handle the situation in such a way that outcomes ensure their self-gains, even if that means inflicting harm on others (Berkowitz and Harmon-Jones, 2004). Human neural systems and hormonal mechanisms facilitate the intense self-protection mode triggered by anger (Harmon-Jones and Sigelman, 2001), exploiting others (Welpe et al., 2012), and deceiving them for personal benefit (Schweitzer and Gibson, 2008).

Further, the self-protective mode activated by performance pressure initiates a cognitive process directed at maximizing self-interest (Mitchell et al., 2018). This phenomenon has been termed as “self-serving cognitions” by researchers. According to Nagin et al. (2002), individuals possessing self-serving cognitions find it completely justified to pursue their personal interest at the expense of others if such a situation arises. Self-serving mindedness rationalizes unethical behavior (e.g., misrepresenting performance) to avoid negative consequences (Reinders Folmer and De Cremer, 2012), which may ensue upon failing to perform as expected. Hence, we propose that performance pressure may lead to workplace cheating behavior due to self-protection needs, anger, and self-serving cognitions.

*Hypothesis 2*: Performance pressure is positively associated with employee cheating behavior.

Based on the proposed relationships between SBLM, performance pressure, and subordinates’ cheating behavior, we expect a mediating effect of performance pressure in the relationship between SBLM and cheating behavior. When organizational situations are exclusively framed as a business problem (i.e., bottom-line mentality) without considering its moral implications, the resulting focus on self-interest may translate into “ethical fading” (Tenbrunsel and Messick, 2004). In pursuit of bottom-line success, high BLM supervisors place excessive performance demands on their subordinates. This constant drive is likely to develop performance pressure among the employees. In response to the discomforting pressure, employees may get involved in workplace cheating behavior in order to ensure their self-interests. Subordinates believe that BLM managers are acting on behalf of the organization when they are constantly exacting high performance levels. Consequently, employees tend to take it out on the organization by engaging in cheating behavior. Hence, we present the following hypothesize:

*Hypothesis 3*: Performance pressure mediates the relationship between SBLM and workplace cheating behavior.

### Negative Reciprocity Beliefs

Even though high SBLM is expected to foster performance pressure among their subordinates, which in turn increases the likelihood of employee cheating behavior, we theorize that the intensity of these effects may vary among employees. Both situational and individual factors influence how strongly SBLM affects subordinate behavior (Farasat et al., 2020). While environmental stimuli may encourage employees to indulge in workplace cheating, their individual moral values and belief systems also play a pivotal role in determining whether or not they engage in unethical work practices (Bratton and Strittmatter, 2013).

Previous researchers have identified a combination of individual characteristics and environmental factors that triggers various forms of unethical conduct among employees. Among the Big Five personality traits, employees with low Conscientiousness and Agreeableness have shown higher levels of organizational deviance (O’Neill et al., 2011). Negative affectivity is another personal attribute that increases the likelihood of engaging in deviant behavior (Aquino et al., 1999). Additionally, individuals with depleted self-regulatory resources (i.e., lower self-control) are expected to involve in unethical conduct (Gino et al., 2011). Interestingly, creative employees tend to be more dishonest as they know how to justify their actions well (Gino and Ariely, 2012). On tasks where workers are expected to achieve stretch goals, instances of cheating greatly increase (Ordóñez et al., 2009). Besides, Liu et al. (2020) explored another type of immoral employee behavior, i.e., unethical pro-family behavior, which is a consequence of moral disengagement induced by high family financial pressure.

Moreover, individuals exhibit more unethical behavior in situations which allow for anonymity (Chen and Wu, 2015), uncertainty and ambiguity (Schweitzer and Hsee, 2002) and where they perceive a lower level of interactional justice (Aquino et al., 1999). Also, people cheat more in order to avoid a loss than to extract a gain (Schindler and Pfattheicher, 2017), or when loss framing is done for piece-rate-based goals (Nagel et al., 2021). In terms of an organization’s ethical climate, employee-centered environments experience lower political deviance (e.g., nepotism, social undermining); a weak focus on rules and regulations leads to higher property deviance (i.e., misuse of organizational assets); and in organizations where employees are highly self-centered, there are greater instances of production deviance (i.e., mild organization-directed deviance; Peterson, 2002). Hence, it can be seen that different types of employee deviance and immoral conduct transpire due to a complex interplay of personal and situational factors.

In this research, we focus on an important individual trait, i.e., negative reciprocity orientation wherein we predict its moderating role in the relationship between BLM, performance pressure, and workplace cheating behavior. Cropanzano and Mitchell (2005) define negative reciprocity as the “tendency to return negative treatment.” According to Gouldner (1960), negative reciprocity is “not the return of benefits but the return of injuries” (p. 172), when an individual is mistreated. Individuals possessing highly negative reciprocity beliefs were found to be angrier along with having a malevolent view of others, which establishes a need for retribution when exposed to undesirable treatment (Eisenberger et al., 2004). Extant research has provided experimental evidence supporting the link between an individual’s reciprocity orientation and their behavioral tendencies (Gallucci and Perugini, 2003). In fact, Mitchell and Ambrose (2007) have asserted that individuals with high negative reciprocity orientation are generally more inclined toward deviant behaviors at the workplace (e.g., theft, shirking responsibility, intentionally prolonging overtime).

Relying on social exchange theory (Blau, 1964), we argue that employees under high levels of performance pressure are expected to cheat as a retaliatory response. However, as individuals have different levels of negative reciprocity beliefs, the impact of a supervisor’s BLM may not be uniformly profound on the workplace cheating behavior of all subordinates. Therefore, the negative outcomes of SBLM might become more pronounced when subordinates attempt retaliation for the unfavorable treatment (i.e., performance pressure) they receive. Specifically, subordinates who strongly endorse negative reciprocity are expected to seek revenge against the behavior of their social exchange partners (Cropanzano and Mitchell, 2005). On the contrary, behaviors like avoidance, reconciliation, or forgiveness are favored by those possessing lower negative reciprocity beliefs (Aquino et al., 2006).

Although negative reciprocity has been established as a *quid pro quo* belief by researchers (Mitchell and Ambrose, 2007), certain circumstances do not allow for it. At such occasions, individuals displace their anger on other more available targets, as explained by the frustration-aggression theory (Dollard et al., 1939). This view suggests that displaced retaliation is a way of catharsis when it is not viable to seek vengeance against the wrongdoer. A meta-analysis of empirical research on human psychology has proven displaced aggression to be a highly intense negative reaction coming from the victim (Marcus-Newhall et al., 2000) Indeed, Ambrose et al. (2002) argue that subordinates may target the organization in an attempt to return the mistreatment of their supervisors. Employees are afraid of their supervisor because of counter-retaliation and the latter’s power and control over rewards, accolades, and punishments (Wang and Noe, 2010). This justifies why they may not consider retribution against their supervisor directly. Hence, displaced aggression could serve to be an excellent alternative for supervisor-targeted retaliation (Jahanzeb et al., 2019).

We have hypothesized that SBLM is responsible for the excessive performance pressure on employees. Frustrated by this pressure, employees with high negative reciprocity may displace their aggression on the organization by engaging in unethical conduct against the organization. Rather than getting back at the supervisor, subordinates vent their frustration on the organization as they perceive it to be less risky, inconspicuous, and wiser. According to Berkowitz (1989), the frequency and intensity of the retaliation increase when there is a higher pressure of attaining a particular goal (i.e., bottom-line success in this case).

Therefore, *an-eye-for-an-eye* strategy or negative reciprocity belief serves as the missing link elucidating when and why some employees cheat as a result of BLM and others do not. Negative reciprocity is a powerful psychological mechanism which even supersedes positive reciprocity (Chernyak et al., 2019), and it significantly affects individual behavior in situations involving economic outcomes (Li et al., 2021), such as bottom-line-driven work environments. In their meta-analysis on 96,930 individuals from 207 studies, Greco et al. (2019) present consistent support for negative reciprocity in explaining why one party shows aggressive behavior (with equal or higher severity and activity) when instigated by negative work behavior from another party in the organization. Along these lines, when subordinates with a high negative reciprocity endorsement are exposed to tremendous work pressure due to BLM of their bosses, their inherent tendency to retaliate is stimulated to an extent that they escalate the reciprocation by hurting the organization through cheating.

Hence, we suggest that the relationship between BLM-induced performance pressure and workplace cheating behavior will be stronger for employees possessing high negative reciprocity. We posit that high (instead of low) levels of negative reciprocity belief reinforce (rather than weaken) the positive relationship between performance pressure and employee cheating. In other words, we state that the indirect effect of SBLM on workplace cheating through performance pressure is dependent on the subordinate’s negative reciprocity belief. Thus, we further present the following hypothesis:

*Hypothesis 4*: Employee negative reciprocity belief moderates the indirect effect of SBLM on employee cheating behavior through performance pressure, such that the indirect effect will be stronger when negative reciprocity belief is higher than when negative reciprocity is lower.

## Overview of Studies

In line with previous studies on BLM (Mawritz et al., 2017; Babalola et al., 2020a, 2022a,b; Quade et al., 2021), and to improve the generalizability of our research findings (Johns, 2006), we tested our hypotheses in two field studies on different populations. Data were collected from Pakistan and United States for Study 1 and Study 2, respectively. Extant research on SBLM has been conducted mostly in the Western context; hence, analyzing the impacts of SBLM on developing economies would serve to further its understanding and enhance its robustness to diverse settings (Babalola et al., 2020a). Using a sample of Pakistani employees in Study 1, we conducted the survey in two time-lagged waves, to examine the mediating role of performance pressure in the relationship between SBLM and employee cheating behavior. Thereafter, for constructive replication, we extended our investigation in Study 2 by incorporating negative reciprocity belief as a second-stage moderator and testing the complete theoretical model using a sample of participants from the United States. Both studies use a similar survey design. Study 2 also expands on Study 1 by demonstrating that the research findings are robust with negative reciprocity belief included in the analysis. Our multisource design shows the generalizability of results and consolidates our contributions to the literature.

As research on BLM is advancing, it shows that this construct is highly generalizable to a wide range of jobs, industrial sectors, and managerial levels (Quade et al., 2021). Investigations have been made in multiple industries including, but not limited to, information technology (Greenbaum et al., 2021); food chain (Lin et al., 2022); banking (Babalola et al., 2020b); financial services (Eissa et al., 2019); and real estate (Mawritz et al., 2017) at different leadership positions. This suggests that BLM is ubiquitous, it exists in all kinds of organizations and can be tested across multiple contexts. Therefore, we collect data from a variety of occupations and companies at varying levels of management.

## Study 1

For testing of the postulated hypotheses, data were gathered from full-time working adults from various firms of Pakistan. Participants filled two time-lagged surveys with a gap of 2 weeks. This time lag was essential for temporal spacing to eliminate or reduce common-method bias (Podsakoff et al., 2012). Additionally, we ensured that the gap is not too long, so as to retain theoretical perspective (George and Jones, 2000). At Time 1, 300 employees were approached to answer questions about demographic information, such as age, gender, education, and time spent with current supervisor. They were also asked to rate their perceptions of SBLM and performance pressure. A total of 300 employees were solicited, and 253 participated in the study (response rate 84.3%). After a gap of 2 weeks (Time 2), these 253 employees were requested to report their cheating behavior along with social desirability. One-to-one matching of responses from Time 1 and Time 2 was carried out. The overall response rate came out to be 71.7% as the final sample comprised valid data from 215 participants.

As shown in Table 1, of the 215 respondents, 146 (67.9%) were male and 49.8% were between 25 and 35 years of age. In terms of education, 57.2% had an undergraduate degree, 33.0% had a Master’s degree, and 1.4% were doctorate; 51.6% of participants were at the middle level of management in their organization. Most of the participants (41.9%) had spent 1 to 2 years with their current supervisor. While the participants represented a wide range of industries (such as biotechnology, pharmaceuticals, food processing, light and heavy engineering, and shoe manufacturing), the highest share (68.4%) came from service industry including software, telecom, and banking sectors.

**Table 1:** Demographic Information (Study 1 and 2).

	Study 1			Study 2	
Variable	Frequency	%	Variable	Frequency	%
*Age*			*Age*		
Less than 25 years	69	32.1	Less than 25 years	6	2.8
25–30 years	107	49.8	25–35 years	115	52.8
31–35 years	22	10.2	36–45 years	65	29.8
36–40 years	9	4.2	46–55 years	22	10.1
41–45 years	2	0.9	More than 55 years	10	4.6
46–50 years	2	0.9			
51–55 years	4	1.9			
*Gender*			*Gender*		
Male	146	67.9	Male	141	64.7
Female	69	32.1	Female	77	35.3
*Qualification*			*Qualification*		
Intermediate	1	0.5	High School	18	8.3
Bachelors	123	57.2	Bachelors	153	70.2
Masters	71	33	Masters	45	20.6
M. Phil	17	7.9	Doctorate	2	0.9
Doctorate	3	1.4			
*Employee Experience*			*Employee Experience*		
Less than 5 years	152	70.7	Less than 5 years	41	18.8
5–10 years	45	20.9	5–10 years	137	62.8
11–15 years	12	5.6	11–15 years	27	12.4
more than 15 years	6	2.8	more than 15 years	13	6
*Organizational Position*			*Organizational Position*		
Entry Level	68	31.6	Entry Level	62	28.4
Middle Level	111	51.6	Middle Level	59	27.1
*Experience under current supervisor*			*Experience under current supervisor*		
Less than 1 year	69	32.1	Less than 1 year	14	6.4
1–2 years	90	41.9	1–2 years	49	22.5
3–5 years	48	22.3	3–5 years	118	54.1
6–10 years	7	3.3	6–10 years	32	14.7
More than 10 years	1	0.5	More than 10 years	5	2.3

### Measures

The scales selected for this survey are based on existing constructs adapted by previous researchers and possess sound psychometric properties. Five-point Likert scale anchors ranging from 1 (“strongly disagree”) to 5 (“strongly agree”) were used.

#### Supervisor Bottom-Line Mentality

Supervisor bottom-line mentality was measured using Greenbaum et al.’s (2012) four-item scale. Example items are “My supervisor is solely concerned about meeting the bottom line” and “My supervisor cares more about profits than his/her employees’ well-being” (1 = “strongly disagree” to 5 = “strongly agree”; Cronbach’s *α* = 0.89, CR = 0.89).

#### Performance Pressure

Participants rated their performance pressure levels using the four-item measure developed by Mitchell et al. (2018). Sample items include “The pressures for performance in my workplace are high” and “I would characterize my workplace as a results-driven environment” (1 = “strongly disagree” to 5 = “strongly agree”; Cronbach’s *α* = 0.87, CR = 0.87).

#### Employee Cheating Behavior

We assessed cheating behavior through the seven-item scale developed by Mitchell et al.’s (2018). Example items are “I made up an excuse to avoid being in trouble for not completing work” and “I lied about the reason I was absent” (1 = “strongly disagree” to 5 = “strongly agree”; Cronbach’s *α* = 0.93, CR = 0.93).

#### Control Variables

Two demographic variables (i.e., age and gender) were used as control variables in this study. According to the previous research (Kish-Gephart et al., 2010), these demographic variables may play a significant role in an individual’s tendency to get involved in unethical workplace behavior. Moreover, we controlled for social desirability bias as impression management can influence the way individuals rate items related to ethical conduct (Randall and Fernandes, 1991). We used an abbreviated 10-item scale (Fischer and Fick, 1993), to gauge an individual’s propensity toward socially desirable responses.

### Validity of Constructs

Prior to hypothesis testing, we carried out confirmatory factor analysis (CFA) with maximum likelihood estimation on SPSS AMOS to determine discriminant validity among the constructs (Anderson and Gerbing, 1988). The results are presented in Table 2. According to the recommendations of Hu and Bentler (1999), normed Chi-square score (*χ*^2^/df) below 3, root-mean-square error of approximation (RMSEA) less than 0.08, and Tucker–Lewis index (TLI) and comparative fit index (CFI) values greater than 0.90 indicate a reasonable fit. It can be observed that the three-factor measurement model, including SBLM, workplace cheating behavior, and performance pressure, has a fairly acceptable fit (*χ*2 = 147.141, df = 87, *χ*^2^/df = 1.694, RMSEA = 0.057, CFI = 0.96, TLI = 0.97). Table 2 demonstrates that the three-factor model has a significantly better fit than the subsequent two-factor and one-factor models. Therefore, we may deduce that the study is not affected by common-method variance (CMV) as the above values represent acceptable discriminant validity. Also, we performed Harman’s one-factor test, and only 33.1% of the variance was explained by the first factor. As this value is less than the 40% threshold recommended by Fuller et al. (2016), CMV concerns are further eliminated.

**Table 2:** Measurement model comparisons (Study 1).

Model	*χ* ^2^	df	Δ*χ*^2^	*χ*^2^/df	RMSEA	TLI	CFI
Three-factor model	147.141^***^	87		1.694	0.057	0.96	0.97
Two-factor model^a^	602.415^***^	89	455.274^***^	6.769	0.164	0.69	0.74
Two-factor model^b^	556.555^***^	89	045.860^***^	6.366	0.158	0.71	0.76
Two-factor model^c^	548.723^***^	89	007.832^***^	6.165	0.155	0.72	0.77
One-factor model	991.942^***^	90	443.219^***^	11.022	0.216	0.46	0.53

p < 0.001 ***.

a *Two-factor model combines SBLM and cheating*.

b *Three-factor model combines SBLM and performance pressure*.

c *Three-factor model combines performance pressure and cheating*.

Further, we checked for individual item reliability—all item loadings were higher than the 0.707 level suggested by Hair et al. (2017). To determine internal consistency, we computed composite reliability (CR). All constructs demonstrated a CR level above the minimum threshold of 0.6 (Tseng et al., 2006). Moreover, convergent validity was established as the average variance extracted (AVE) values for all constructs were higher than 0.5 (Bagozzi and Yi, 1988). Discriminant validity was validated as the square root of AVE values for the constructs (shown in Table 3) exceeded their respective correlations with other constructs in the study (Fornell and Larcker, 1981). Finally, all measures achieved acceptable levels of Cronbach’s alpha (higher than 0.7) showing sufficient internal reliability (Nunnally, 1994). To rule out the issue of multicollinearity, we computed the variance inflation factor (VIF) of our regression coefficients. All VIF scores were less than 10 (the highest being 1.102), indicating that multicollinearity did not raise a biasing concern (Aiken et al., 1991).

**Table 3:** Descriptive statistics, correlations and AVE Values (Study 1).

Variable	Mean	SD	1	2	3	4	5	6
1. SBLM	3.05	0.85	**(0.81)**					
2. Performance pressure	2.99	0.88	0.25^**^	**(0.80)**				
3. Cheating	2.19	0.77	0.16^*^	0.23^**^	**(0.80)**			
4. Social desirability bias	3.42	0.56	−0.18^**^	−0.04	−0.40^**^			
5. Age^a^	2.02	1.14	0.06	−0.04	−0.08	−0.07	–	
6. Gender^b^	0.32	0.47	−0.15^*^	−0.02	−0.05	−0.04	−0.18^**^	–

*N* = 215. Diagonal shows square root of AVE. ^*^p < 0.05, ^**^p < 0.01.

a Age was measured using an 8-point scale (where 1 = “<25 years,” 2 = “25–30 years,” 3 = “31–35 years,” 4 = “36–40 years,” 5 = “41–45 years,” 6 = “46–50 years,” 7 = “51–55 years,” 8 = “>56 years”).

b 0 = male, 1 = female.Diagonal shows square root of AVE values in bold.

### Descriptive Statistics and Correlations

Descriptive statistics, zero-order correlations, and square root of AVE values for the study variables are summarized in Table 3.

### Hypothesis Testing

For hypothesis testing, we utilized Hayes (2017) PROCESS macro in SPSS to estimate the mediation effects. Researchers have extensively used this procedure to determine direct, indirect, and conditional indirect effects with relevant bootstrapped confidence intervals. It helps in overcoming statistical power issues that may arise due to data asymmetric or non-normal sampling distributions of indirect relationships (MacKinnon et al., 2004; Hayes, 2017).

PROCESS macro Model 4 of SPSS was used to examine mediation, in which we evaluated the indirect effect of SBLM on employee cheating behavior through performance pressure (Hypothesis 3). For indirect effects, the bootstrapping method with 5,000 resamples was used—it results in 95% bias-corrected confidence intervals (CI). Hayes (2017) suggests that a significant indirect effect exists if zero is not included in the confidence interval.

Hypothesis 1 states that a positive association exists between SBLM and performance pressure. As shown in Table 4, SBLM has a significant positive association with performance pressure (*β* = 0.27, SE = 0.07, *p* < 0.01). Hypothesis 2 is also supported, wherein performance pressure positively predicts workplace cheating behavior among employees (*β* = 0.17, SE = 0.06, *p* < 0.01 Therefore, hypotheses 1 and 2 are accepted.

**Table 4:** Regression results for performance pressure and cheating behavior (Study 1).

Variables	Performance pressure			Cheating behavior		
Control	Model 1			Model 2		
	B	SE	95% CI	B	SE	95% CI
Age	−0.04	0.05	[−0.15, 0.06]	−0.07	0.04	[−0.16, 0.01]
Gender	0.03	0.13	[−0.23, 0.28]	−0.02	0.10	[−0.22, 0.19]
Social desirability bias	0.01	0.11	[−0.20, 0.22]	−0.53^**^	0.09	[−0.70, −0.36]
SBLM	0.27^**^	0.07	[0.12, 0.41]	0.04	0.06	[−0.08, 0.16]
Performance pressure				0.17^**^	0.06	[0.06, 0.28]
*R* ^2^	0.07			0.21		
	*F* (4, 210) = 3.72, *p* < 0.001			*F* (5, 209) = 11.38, *p* < 0.001		

For Hypothesis 3, we tested the mediating role of performance pressure in the relationship between SBLM and employee cheating behavior. Results from the application of SPSS PROCESS Model 4 indicate that zero is excluded from the CI (*β* = 0.05 (0.02), 95% CI = 0.01, 0.10). Thus, Hypothesis 3 is validated. Altogether, Study 1 results demonstrated a statistically significant positive indirect effect of SBLM through performance pressure on cheating behavior.

## Study 2

For data collection of Study 2, we recruited 300 employees from the United States through Amazon Mechanical Turk (MTurk) in a two-wave study. This platform was selected because it has a swift, streamlined process, which helps in obtaining a more demographically diverse sample than traditional methods while maintaining similar reliability levels (Buhrmester et al., 2016). To ensure high-quality data, we adopted best practices such as including high reputation workers (with above 95% acceptance rate; Peer et al. (2014)) and those who have previously completed more than 100 human intelligence tasks (HITs). Moreover, based on the recommendations of Aguinis et al. (2021), we collected data from an additional 20% of MTurkers to allow for participant attrition. We paid 1 USD for one completed survey.

Participants filled two time-lagged surveys with a gap of 2 weeks. At Time 1, 300 MTurk workers were recruited to share their demographic information (e.g., age, gender, education, time spent with current supervisor, etc.) along with unique MTurk Worker IDs. At the same time, data related to SBLM, performance pressure, and negative reciprocity beliefs were gathered. When 300 responses were completed, they were screened for inattentiveness checks discarding 11 responses (response rate 96.3%). At Time 2, 2 weeks later, we used the collected MTurk Worker IDs to approach the remaining 289 employees for second wave of data collection. Participants answered questions related to workplace cheating behavior and social desirability. After one-to-one matching of MTurk IDs, the final sample comprised 218 valid responses, resulting in an overall response rate of 72.7%.

As shown in Table 2, of the 218 participants, 141 (64.7%) were male and 52.8% were between 25 and 35 years of age. In terms of education, 70.2% had an undergraduate degree, 20.6% had a Master’s degree, and 0.9% were doctorate; 62.8% of participants had an experience of 5 to 10 years with their current organization. Most of the participants (54.1%) had spent 3–5 years with their current supervisor. While the participants represented a wide range of industries (such as agriculture, fertilizer and chemicals, heavy engineering, pharmaceuticals, and textile), the highest share (40.4%) came from the service industry including software, telecom, and banking sectors.

### Measures

For Study 2, we used similar scales as Study 1 for BLM (Cronbach’s *α* = 0.82, CR = 0.82), performance pressure (Cronbach’s *α* = 0.79, CR = 0.79), and workplace cheating behavior (Cronbach’s *α* = 0.91, CR = 0.91).

#### Negative Reciprocity Beliefs

Negative reciprocity beliefs were evaluated with a 14-item scale developed by Eisenberger et al. (2004). Sample statements include “If someone dislikes me, I should dislike them” and “I should not give help to those who treat me badly” (1 = “strongly disagree” to 5 = “strongly agree”; Cronbach’s *α* = 0.96, CR = 0.96).

#### Control Variables

Following Study 1, we controlled for age, gender, and social desirability. Based on recommendations of Valentine and Fleischman (2018), we used two items from the abbreviated 10-item scale (Fischer and Fick, 1993), to gauge an individual’s propensity toward socially desirable responses. The items are “I’m always willing to admit it when I make a mistake” and “I always try to practice what I preach” (1 = “strongly disagree” to 5 = “strongly agree”).

### Validity of Constructs

The results of CFA are presented in Table 4. It can be observed that the four-factor measurement model, including SBLM, employee cheating behavior, performance pressure, and negative reciprocity beliefs, has a fairly acceptable fit (*χ*^2^ = 536.841, df = 318, *χ*^2^/df = 1.688, RMSEA = 0.056, CFI = 0.94, TLI = 0.94). Table 5 reveals that the four-factor model has a significantly better fit than the three-factor and one-factor models. Therefore, discriminant validity is established. In the Harman’s one-factor test, a single factor extracted 44.5% of the variance, which is below the 50% criterion suggested by Podsakoff and Organ (1986). Hence, there exists no major issue of common-method bias.

**Table 5:** Measurement model comparisons (Study 2).

Model	*χ* ^2^	df	**Δ** *χ* ^2^	*χ*^2^/df	RMSEA	TLI	CFI
Four-factor model	536.841^***^	318		1.688	0.056	0.94	0.94
Three-factor model^a^	681.996^***^	321	145.155^***^	2.125	0.072	0.90	0.91
Three-factor model^b^	731.721^***^	321	049.725^***^	2.280	0.077	0.89	0.89
Three-factor model^c^	740.172^***^	321	008.451^***^	2.306	0.078	0.88	0.89
Three-factor model^d^	984.093^***^	321	243.921^***^	3.066	0.098	0.81	0.83
One-factor model	1248.915^***^	324	264.822^***^	3.855	0.115	0.74	0.76

^***^p < 0.001.

a Three-factor model combines SBLM and cheating.

b Three-factor model combines SBLM and performance pressure.

c Three-factor model combines performance pressure and cheating.

d Three-factor model combines negative reciprocity and cheating.

We examined individual item reliability—most of the item loadings were greater than the 0.707 threshold (Hair et al., 2017). Only two items of the negative reciprocity measure (items 7 and 13 which were reverse-coded) showed insufficient loadings (less than 0.3) and were dropped following the example of Al Halbusi et al. (2021), as they affect the measurement quality of their corresponding first- or second-order constructs (Hair et al., 2017). Also, the constructs of Study 2 fulfilled the above-mentioned criteria for internal consistency (CR > 0.6) and internal reliability (Cronbach’s *α* > 0.7). In line with the recommendations of Huang et al. (2013), convergent validity was achieved as AVE values of the constructs exceeded 0.4 (Table 6). Discriminant validity was attained as the constructs’ square roots of AVE values were greater than their inter-item correlations (Hair et al., 2017). Multicollinearity was also not a concern as all VIF values were below 10 (the highest being 1.910).

**Table 6:** Descriptive Statistics, Correlations, and AVE Values (Study 2).

Variable	Mean	SD	1	2	3	4	5	6	7
1. SBLM	3.60	0.90	**(0.73)**						
2. Performance pressure	3.49	0.88	0.37^**^	**(0.70)**					
3. Negative reciprocity	3.10	1.06	0.50^**^	0.63^**^	**(0.80)**				
4. Cheating	3.13	1.07	0.57^**^	0.42^**^	0.63^**^	**(0.77)**			
5. Social desirability bias	3.87	0.87	−0.15^*^	0.14^*^	0.00	−0.09	–		
6. Age^a^	2.61	0.88	−0.02	0.05	−0.01	−0.09	0.10	–	
7. Gender^b^	1.35	0.48	0.10	0.12	0.01	0.07	0.02	0.21^**^	–

N = 218. Diagonal shows square root of AVE. ^*^p < 0.05, ^**^p < 0.01; ^a^Age was measured using a 5-point scale (where 1 = “< 25 years,” 2 = “25–35 years,” 3 = “36–45 years,” 4 = “46–55 years,” 5 = “> 55 years”). ^b^1 = male, 2 = female.Diagonal shows square root of AVE values in bold.

### Descriptive Statistics and Correlations

Descriptive statistics and zero-order correlations among the study variables are summarized in Table 6.

### Hypothesis Testing

In study 2, we test the complete theoretical framework that represents a second-stage moderated mediation. For mediation, we use the procedure similar to Study 1. Further, Model 14 of SPSS PROCESS macro was used to assess the moderating role of negative reciprocity belief in the relationship between performance pressure and employee cheating behavior (Hypothesis 4), and the moderated mediation where negative reciprocity moderates the indirect effect of SBLM on cheating through performance pressure (Hypothesis 5). To evaluate moderated mediation, we computed conditional indirect effects at three levels of negative reciprocity belief (i.e., mean, one standard deviation above and one standard below the mean) by using 5,000 bootstrapped samples for hypothesis testing (Edwards and Lambert, 2007). The results are shown in Tables 4, 5.

Table 7 reveals that Hypothesis 1 is accepted (*β* = 0.38, SE = 0.06, *p* < 0.01), wherein SBLM positively influences performance pressure. Hypothesis 2 is also validated (*β* = 0.33, SE = 0.06, *p* < 0.01), showing a significant positive association between performance pressure and employee cheating behavior.

**Table 7:** Regression results for performance pressure and cheating (Study 2).

Variables	Performance pressure			Cheating		
Control	Model 1			Model 2		
	B	SE	95% CI	B	SE	95% CI
Age	−0.12	0.07	[−0.25, 0.02]	−0.11	0.06	[−0.23, 0.01]
Gender	0.04	0.12	[−0.20, 0.28]	0.12	0.11	[−0.10, 0.34]
Social desirability bias	−0.06	0.07	[−0.19, 0.08]	−0.08	0.06	[−0.20, 0.05]
SBLM	0.54^**^	0.07	[0.41, 0.68]	0.34^**^	0.07	[0.20, 0.57]
Performance pressure				0.12	0.09	[−0.05, 0.29]
Negative reciprocity				0.43^**^	0.07	[0.30, 0.57]
Performance pressure x Negative reciprocity				0.12^*^	0.06	[0.01, 0.23]
*R* ^2^	0.39			0.50		
	*F* (5, 212) = 27.08, *p* < 0.001			*F* (7, 211) = 29.94, *p* < 0.001		

For Hypothesis 3, we tested the mediating effect of performance pressure in the association between SBLM and employee cheating behavior. Results from the application of SPSS PROCESS Model 4 indicate that zero is excluded from the CI (*β* = 0.12 (0.04), 95% CI = 0.07, 0.21). Thus, Hypothesis 3 is validated. It can be seen that results from Study 1 (Hypotheses 1–3) are reinforced by Study 2.

**Figure 2:**
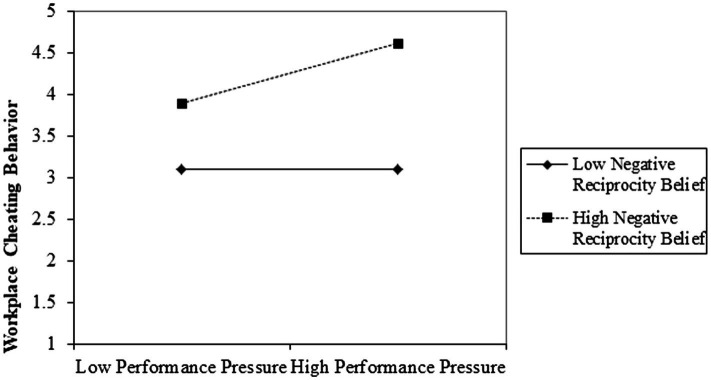
The moderating effect of negative reciprocity on the relationship between performance pressure and employee cheating.

Hypothesis 4 posits that employee negative reciprocity moderates the indirect effect of SBLM on employee cheating behavior through performance pressure. For testing the hypothesis, we used 95% bias-corrected CI with bootstrapping (5,000 samples) in Hayes (2017) PROCESS model 14 for the conditional indirect effect at three levels (mean, +1 SD, −1 SD) of negative reciprocity. Table 8 shows that the conditional indirect effect (SBLM → performance pressure → workplace cheating behavior) is significant (zero is not included in CI) when subordinates have high (+1 SD) levels of negative reciprocity (*β* = 0.095 (0.052), 95% CI = 0.004, 0.210). Further, the interaction at ± SD of negative reciprocity was plotted (Figure 2) to evaluate the invigorating effect of negative reciprocity on performance pressure in increasing workplace cheating behavior. Hence, Hypothesis 4 is accepted.

**Table 8:** Conditional indirect effect of SBLM on cheating through performance pressure.

Levels of negative reciprocity	Effect	SE	Boot *LL*CI	Boot *UL*CI
−1 *SD* (−1.06)	−0.004	0.036	−0.073	0.069
M (0)	0.045	0.038	−0.023	0.126
+1 *SD* (1.06)	0.095	0.052	0.004	0.210

## Discussion

This study evaluates the mediating effect of performance pressure on the relationship between SBLM and cheating while also highlighting the critical moderating role of negative reciprocity in the process. We found empirical evidence for a moderated mediation framework in which performance pressure mediated the relationship between SBLM and cheating, and negative reciprocity belief moderated this relationship at the second stage. Our findings generally support previous literature, which suggests a positive impact of leader’s bottom-line approach on unethical behavior by employees (Mesdaghinia et al., 2019; Babalola et al., 2020a, 2021; Zhang et al., 2021). In particular, we demonstrate that high SBLM leads to increase in subordinate cheating, thereby reinforcing the argument by Farasat et al. (2021). They have theorized that organizational managers’ bottom-line thinking triggers workaholism among their workers, which eventually causes them to cheat, especially when they feel psychologically entitled. Furthermore, our study provides evidence that performance pressure leads to cheating behavior—this is in line with prior research on performance pressure (Mitchell et al., 2018). Individuals under performance pressure experience anger due to which they may enter into a self-protective mode and these self-serving cognitions cause them to cheat for self-gain.

Several studies have shown that high negative reciprocity endorsement contributes in exacerbating undesirable conduct by employees (Wang, 2011; Wu et al., 2014; Faldetta, 2020)—we have further established this role of negative reciprocity in moderating the relationship between SBLM, performance pressure, and cheating. Subordinates tend to experience undue pressure due to excessive bottom-line demands of their bosses and those with high negative reciprocity orientation retaliate by harming the organization (i.e., cheating) through displaced aggression. This is consistent with prior research findings, where displaced retaliation combined with negative reciprocity strengthens the relationship between supervisor mistreatment (i.e., abusive supervision) and dysfunctional employee conduct like workplace deviance (Mitchell and Ambrose, 2007) and knowledge hiding (Jahanzeb et al., 2019). As the integration of negative reciprocity in BLM research is a new concept, further research is warranted on how and when it may affect other types of negative behaviors by employees.

### Theoretical Contributions

Our study has significant theoretical contributions. Emerging literature on SBLM suggests that it precipitates into unethical behavior among subordinates (Mesdaghinia et al., 2019; Farasat et al., 2020; Zhang et al., 2020). Yet, to date, there is limited understanding about the cognitive mechanisms that may explain these undesirable consequences. We identify performance pressure as an integral link through which high BLM supervisors unintentionally motivate workplace cheating behavior.

Additionally, our research highlights the moderating role of negative reciprocity belief in the link between SBLM, performance pressure, and cheating. As a second-stage moderator, negative reciprocity has shown to exacerbate the positive impact of BLM and performance pressure on cheating behavior. Previous research has suggested that BLM has both functional and dysfunctional consequences (Quade et al., 2021). As the extent of vengeance is dependent on individual differences in negative reciprocity belief (Eisenberger et al., 2004), it may explain why employees respond to SBLM differently. Therefore, negative reciprocity belief serves as a critical boundary condition, which can invigorate the negative effects of BLM on organizational outcomes. This is a major contribution of our research as it is the first study to relate negative reciprocity belief and displaced retaliation to BLM and its effects.

Finally, previous literature on BLM has mostly tested its effects in developed economies, for instance United States. We test our theoretical model on two different populations, where one is developed (United States) and the other is developing (Pakistan). Also, American culture is more individualistic in nature, while that of Pakistan is primarily collectivistic. Such differences in cultures necessitate a deeper understanding of how social exchanges in response to high BLM may vary in these contexts. Individuals from collectivist societies possess a higher reciprocity orientation than their counterparts from individualistic societies (Shen et al., 2011). However, results from the current analyses reveal that the relationship between BLM and workplace cheating is almost similar across Pakistani and American cultures. This shows that BLM and its impacts are not affected by the individualism–collectivism dimension of culture. In this regard, our findings are line with Babalola et al. (2020a), as they also demonstrate the robustness of BLM impacts to multiple contexts: Nigeria (a developing society) and China (an industrialized society). Hence, our work advances this emerging line of inquiry.

### Practical Implications

Our study offers a number of managerial implications for organizations desirous of maximizing their profitability whilst establishing a strong ethical climate. It should be recognized that bottom-line mentality has beneficial as well as detrimental workplace outcomes. As such, it is essential to assimilate our findings into leadership training programs to facilitate leaders in adopting a more balanced supervisory approach, keeping in mind how BLM affects subordinates’ behavior. Moreover, it is also advisable to devise recruitment and selection processes in such a way that candidates with a multidimensional personality—focusing on social, ethical, and environmental considerations along with financial ones—are hired (Eissa et al., 2019). Organizational recognition, rewards, and punishment should include strict criteria related to moral reasoning and ethical behavior, besides productivity and profitability. Finally, employee training and development initiatives should focus on moral education in an attempt to limit cheating and similar unethical behaviors. All these measures help in the institutionalization of ethics in organizations, which in turn minimizes the development of BLM among leaders.

Our findings imply that performance pressure motivates counterproductive work behavior, i.e., cheating. Therefore, BLM supervisors should take caution when setting unrealistically high-performance targets for their subordinates. Incorporating incentives for ethical conduct into the performance-related goals could help in curbing temptations to cheat. Additionally, a robust accountability policy, code of ethics, and strong monitoring systems could further decrease the frequency of workplace cheating incidents as part of fulfilling performance expectations (Nagel et al., 2020). High-performance demands should be communicated so as to emphasize the fundamental importance of adhering to ethical and legal standards alongside pursuit of bottom-line attainment. Further, it would help to conduct workshops and counseling sessions for employees to equip them with coping strategies to mitigate the amount of performance pressure they experience. A more balanced self-affirmation approach may be particularly useful in counteracting the impact of performance pressure on cheating (without dispelling its benefits), wherein employees make conscious efforts to reflect on their core personal values (Spoelma, 2021). This would enable them to effectively deal with pressure-induced anger and self-serving cognitions, rather than acting defensively in self-interest.

The current work has underscored the pivotal significance of negative reciprocity belief in amplifying the impact of SBLM (coupled with performance pressure) on cheating behavior. Use of questionnaires and vignettes could prove to be helpful in determining the negative reciprocity orientation of employees. If it is found high, organizations could benefit by apprising them of the deleterious effects of the tit-for-tat spiral and advising them to consider alternative approaches such as forgiveness, negotiation, avoidance, and clarity-seeking (Aquino et al., 2006). Furthermore, training employees to thoroughly analyze the situation before jumping toward strong retribution may serve to minimize the hostile feelings responsible for unethical conduct. Moreover, as negative reciprocity is strongly associated with trait anger (Eisenberger et al., 2004), employees should be regularly reminded of anger management techniques (e.g., deep breathing, taking time out) so that they do not react indignantly in high-pressure situations. Finally, it is advisable for supervisors and subordinates to nurture a positive social exchange relationship with trust, mutual interest, and openness as its essential ingredients to reduce instances of negative reciprocity.

### Limitations and Future Directions

Although our research has several strengths, we acknowledge that it has its share of limitations. First, data are collected from a single source (employees), which may raise concerns about common-method bias. However, we ensured temporal spacing in gathering data for predictor and outcome variables. This helps in decreasing consistency motif, which is one of the main causes of CMV (Podsakoff et al., 2012). Additionally, we carried out statistical analysis to rule out CMV issue in our study’s design. In CFA, our three-factor (Study 1) and four-factor (Study 2) models had a significantly better fit than other statistical model alternatives, showing adequate discriminant validity. Nevertheless, future research could use a multisource (such as supervisor’s assessment, co-workers’ assessment, direct-observation) design and greater time lag between surveys for more robust inferences.

Moreover, our research design was cross-sectional, which makes it difficult to guarantee a causal relationship between variables studied despite the temporal spacing. We did take caution to align the causal sequencing of our conceptualized model with theoretical and practical standpoints. Even so, it would be beneficial for future studies to conduct longitudinal and experimental analyses to provide greater credence to causality. Further, the use of self-report measures for sensitive data (that is deviance and/or unethical conduct) is a limitation as employees may underreport such behavior due to the fear of disciplinary action if caught. To deal with this, the respondents were assured confidentiality and anonymity to reduce potential uneasiness that may arise upon rating one’s ethicality. Nonetheless, objective data could be incorporated for higher quality of results.

Further, our investigation deliberated on the focal role of performance pressure as an explanatory mechanism that underpins the negative effects of BLM on unethical behavior. Future studies could evaluate a mediating role of other variables like supervisor close monitoring and contingent rewards/punishments to determine how the dysfunctional consequences of SBLM can be mitigated and positive outcomes be projected. Besides, since performance pressure is a discomforting feeling, it could be deeply insightful to examine its combined effect with BLM on employee psychological and physical well-being in future researches.

Additionally, the current work investigates negative reciprocity as the primary boundary condition that reinforces the indirect link between SBLM and cheating behavior. However, there can be a multitude of other contingency factors that may be considered. Exploring which personality traits are responsible for different types of cheating behavior could open up interesting arenas for future research. Evaluations of an organization’s tolerance to deviance could provide a deeper understanding on how the external environment influences the dysfunctional effects of SBLM. Furthermore, future studies could also focus on the identification of antecedents of BLM to understand the interplay of factors that are responsible for the phenomenon at hand.

## Conclusion

In this enquiry, we rely on social exchange theory along with displaced aggression to gain a nuanced understanding of the mechanism through which managers’ bottom-line mentality stimulates unethical behavior among subordinates. In particular, we highlight the crucial role of negative reciprocity orientation in explaining why employees respond differently to same levels of SBLM. We found that high-BLM supervisors induce performance pressure among their subordinates, which increases the likelihood of workplace cheating behavior. Further, we demonstrate that this process is exacerbated by high negative reciprocity belief of the employees. Based on these findings, future research investigating ways to optimize SBLM, reduce performance pressure, and manage negative reciprocity is necessary.

## Data Availability Statement

The raw data supporting the conclusions of this article will be made available by the authors, without undue reservation.

## Ethics Statement

The studies involving human participants were reviewed and approved by Ethics Committee on research involving human participants for FAST School of Management, National University of Computer & Emerging Sciences, Lahore. The patients/participants provided their written informed consent to participate in this study.

## Author Contributions

All authors listed have made a substantial, direct, and intellectual contribution to the work and approved it for publication.

## Conflict of Interest

The authors declare that the research was conducted in the absence of any commercial or financial relationships that could be construed as a potential conflict of interest.

## Publisher’s Note

All claims expressed in this article are solely those of the authors and do not necessarily represent those of their affiliated organizations, or those of the publisher, the editors and the reviewers. Any product that may be evaluated in this article, or claim that may be made by its manufacturer, is not guaranteed or endorsed by the publisher.
